# Effect of Shi-Zhen-An-Shen herbal formula granule in the treatment of young people at ultra-high risk for psychosis: a pilot study

**DOI:** 10.3389/fpsyt.2023.1160452

**Published:** 2023-06-27

**Authors:** Hong Zhu, Yanzhe Ning, Sisi Zheng, Sitong Feng, Linrui Dong, Hongxiao Jia

**Affiliations:** ^1^Beijing Key Laboratory of Mental Disorders, National Clinical Research Center for Mental Disorders & National Center for Mental Disorders, Beijing Anding Hospital, Capital Medical University, Beijing, China; ^2^Advanced Innovation Center for Human Brain Protection, Capital Medical University, Beijing, China

**Keywords:** ultra-high risk, Chinese herbal medicine, cognitive impairment, social functioning, schizophrenia

## Abstract

**Introduction:**

To date, there is no conclusive evidence for early interventions on ultra-high risk (UHR) for psychosis. The Chinese herbal medicine is confirmed to be beneficial in improving psychiatric symptoms and cognitive impairments for schizophrenia patients. However, the effect of Chinese herbal medicine on treating UHR patients remains unknown.

**Methods:**

Eighty UHR patients were recruited from the outpatient department. They were randomly assigned to receive either Shi-Zhen-An-Shen herbal formula granule (SZAS-HFG) combined with aripiprazole placebo or aripiprazole combined with SZAS-HFG placebo for a 12-week treatment. The psychiatric symptoms were assessed using the Structured Interview for Prodromal Syndromes (SIPS). The Trail Making Test part A (TMT-A), Brief Visuospatial Memory Test (BVMT), Hopkins Verbal Learning Test (HVLT), and Continuous Performance Test (CPT) were used to assess cognitive functions. we also employed the Global Assessment of Functioning (GAF) to evaluate social functioning. The linear mixed-effects models were performed to detect the difference in effectiveness between the two groups.

**Results:**

After 12-week treatment, both groups showed significant effects of time on SIPS, TMT-A, HVLT, BVMT, and GAF. There was a significant effect of group only on CPT. Moreover, we also found a significant interaction effect on GAF.

**Conclusion:**

SZAS-HFG can effectively alleviate psychosis symptoms, and improve cognitive impairments and overall functioning as well as aripiprazole.

**Clinical trial registration:** Chinese Clinical Trial Registry, ChiCTR-IOR-17013513.

## 1. Introduction

Schizophrenia is a severe chronic psychotic disorder with substantial disability, morbidity, mortality, and a heavy burden on family and society (1). A clinical staging model of psychiatric disorders has been proposed to seek for an earlier and more effective intervention (2, 3). During this clinical staging model, ultra-high risk (UHR) subjects are focused, who experience some psychotic symptoms and cognitive deficits (4). Notably, cognitive impairments are the core early features of UHR patients (5). It has been reported that 20% after 1 year and an average 35% over 3 years of UHR subjects convert into diagnosable psychosis (6). It is essential to take adequate measures for patients at the stage of UHR. Early detection and effective treatment are two main elements of early intervention in schizophrenia (7). The UHR criteria are validated at present, which can be applied for evaluating treatment strategies to improve social function, relieve symptoms, and reduce the transition rate (1, 8–11). In hence, it is the right time to draw more attention on seeking a more effective and safe therapy for UHR subjects.

At the stage of UHR, cognitive behavioral therapy (CBT), antipsychotic drugs and ω-3 polyunsaturated fatty acids alone or in combination have been applied to prevent the onset of psychosis (12–16). The study on Chinese UHR patients has shown a higher conversion rate in patients receiving antipsychotics than those who do not (17). The CBT and long-chain ω-3 polyunsaturated fatty acids, are considered safer interventions and the preferred options for first-line treatment. Nevertheless, there is no significant difference in transition rates between ω-3 polyunsaturated fatty acids group and placebo group in a multi-center trial (18). Moreover, the Cochrane Database systemic review has shown no conclusive evidence for above early interventions on UHR subjects (7). It is necessary to seek another adequate treatment to prevent psychosis in patients at UHR.

As the main element of traditional Chinese medicine (TCM), both the Chinese herbal medicine and acupuncture have gained worldwide recognition, and have been applied during clinical practice (19, 20). Furthermore, clinical trials and systemic reviews have suggested that the Chinese herbal medicine as an additional therapy may be beneficial in improving treatment efficacy and reducing adverse events for schizophrenia (21–24). Notably, several animal studies have reported that some active ingredients of Chinese herbal medicine, cornel iridoid glycoside and tetrahydroxystilbene glucoside have divergent therapeutic effects on cognitive impairments and neurological defects (25, 26). However, to our knowledge, there is no clinical trial to explore the efficacy of Chinese herbal medicine in treating young people at UHR for psychosis.

Given that the Chinese herbal medicine is beneficial in improving treatment efficacy for schizophrenia patients, we hypothesized that the Chinese herbal medicine was not inferior to aripiprazole on treating UHR patients. In the current study, we design a prospective, double-blind, placebo-controlled, randomized study. We choose the Shi-Zhen-An-Shen herbal formula granule (SZAS-HFG) as the treatment of drug and aripiprazole as the positive control, to evaluate the efficacy of Chinese herbal medicine in relieving psychiatric symptoms and improving cognitive functions.

## 2. Methods

### 2.1. Participants

The current study was approved by the Beijing Anding Hospital Ethics Committee. All subjects signed informed consents before enrolling in this study. The 80 UHR patients were recruited from December 2017 to November 2020 at the outpatient department for UHR in Beijing Anding Hospital. Subjects would be allowed to participate in our trial if they were aged from 16 to 30 years, signed consent and met the UHR criteria, which was defined via the Chinese version of Structured Interview for Prodromal Syndromes (SIPS) with good interrater reliability and validity by a well-trained interviewer (17). We recruited two subtypes according to the UHR criteria, including attenuated positive symptom syndrome (APSS), and brief intermittent psychotic syndrome (BIPS). Moreover, all recruited participants should also met the criteria of the standard of TCM of hyperactivity of fire due to deficiency of kidney yin (soreness and weakness of waist and knees, dry mouth, red tongue, thin tongue coating with teeth marks, and fine rapid stringlike pulse).

Subjects would be ruled out if they meet any criteria below: (i) with a history of serious or unstable somatic diseases; (ii) with antipsychotic medications within 4 weeks before recruitment; (iii) unattended or unable to take medicine as directed by a doctor; (iv) with severe suicidal tendencies; (v) compared with the screening period, the reduction rate of SIPS at the baseline over 25% after 1 day.

### 2.2. Study design and randomization

A single-center, prospective, double-blind, randomized, placebo-controlled clinical trial was conducted. Eligible patients were assigned to the SZAS-HFG group or control group after a computerized completely randomization. The computer-generated random sequence was administered by an independent third party until all study data were completed. All participants and those involved in administering interventions, assessing outcomes, data entry, and data analyses were blind to group assignment. To test effectiveness of blinding, a questionnaire would be acquired from participants at week 12.

### 2.3. Study intervention

The patients in SZAS-HFG group were treated with SZAS-HFG and aripiprazole placebo, while patients in the control group received aripiprazole and SZAS-HFG placebo. The aripiprazole (5 mg/tablet) and placebo tablet were provided by Zhejiang Otsuka Pharmaceutical Co, Ltd. The SZAS-HFG and placebo were produced by Beijing Kang Ren Tang Pharmaceutical Co, Ltd., China. The SZAS-HFG was composed of Chrysanthemum, *Cornus officinalis*, dried Rehmannia root, Turmeric, the root of Fangfeng and *Polygonum multiflorum*. The placebos in our study were the same in smell, taste, color, appearance, packaging, and tag as SZAS-HFG or aripiprazole.

The aripiprazole or placebo started with 2.5 mg per day, and an increase of 5 mg every 1–2 days to the maximum dose of 5–10 mg per day. Patients dissolved granules into 100 mL boiled water, and then took it between 30 and 36°C. The SZAS-HFG or placebo would be taken in one bag per day. The entire course of treatment lasted 12 weeks. Both groups were permitted to use trihexyphenidyl 2–4 mg/day for treating extrapyramidal reactions induced by aripiprazole. Zopiclone and zolpidem were allowed to treat insomnia, continuously less than 7 days.

### 2.4. Assessments

Psychosis symptoms were assessed using the Scale of Prodromal Syndromes (SOPS) from the SIPS interview tool at baseline, week 4, and week 12, which was set as the primary outcome (20).

The assessments of cognitive functions were also set as the primary outcome. Prior to the neuropsychological tests, the Wechsler Intelligence Scale for Adults was applied to evaluate intelligence quotient. The cognitive assessments lasted 1 h and were performed following the order below: Trail Making Test part A (TMT-A) assessing information processing speed and executive function, Hopkins Verbal Learning Test (HVLT, Chinese version) assessing verbal learning, Brief Visuospatial Memory Test (BVMT) assessing visual learning, and Continuous Performance Test (CPT) assessing attention. Moreover, during HVLT and BMVT, the delayed recall was set as the primary outcome, and alternative forms was applied for retest.

The assessment of psychological, social and occupational functioning was set as the secondary outcome and measured by the Global Assessment of Functioning (GAF), a scale from 1 to 100. The low score represented worse overall functioning.

### 2.5. Estimated of required sample size

Based on the score of SOPS revealed by the randomized trial of olanzapine on UHR patients (27), the standard deviation of the control group was 16.7, and the SZAS-HFG group was set to 15.1. A non-inferiority analysis was conducted with PASS Software version 15.0.5^1^ using an alpha of 0.025, a power of 0.8, and margin of non-inferiority (10.7) to determine the sample size. The true difference between the means was assumed to be 0.0. The sample size was estimated 36 at each group. Considering a dropout rate of 10%, a total of 80 participants were finally required.

### 2.6. Patient safety

Adverse events referred to any medical event related with taking SZAS-HFG or aripiprazole. We recorded the adverse events related to medication at each visit. Meanwhile, the psychiatrist conducted active management of adverse reactions for the patients.

### 2.7. Data analysis

We performed data analysis using SAS software (9.4 version, SAS Institute, Cary, NC). Continuous data were shown with mean ± standard deviation, and discrete data were displayed with constituent ratio. The independent *t*-test or nonparametric Mann–Whitney test was utilized to assess normality of the data between the two groups at baseline.

The intention-to-treat (ITT) analysis was conducted for the primary outcome. The recruited participants were included in the full analysis set. The three time points (baseline, week 4 and week 12) were considered as a four-level repeated measure during data analysis. The mixed-model analysis was employed to calculate the main effects of time point, group, and the interaction between group and time point. The missing data were estimated using the multiple imputation approach in this study. Effect sizes (Hedges’ *g* scores) were calculated for post-treatment scores, in comparison with the baseline. We set *p* < 0.05 as the significant level.

## 3. Results

### 3.1. Recruitment

**Figure 1:**
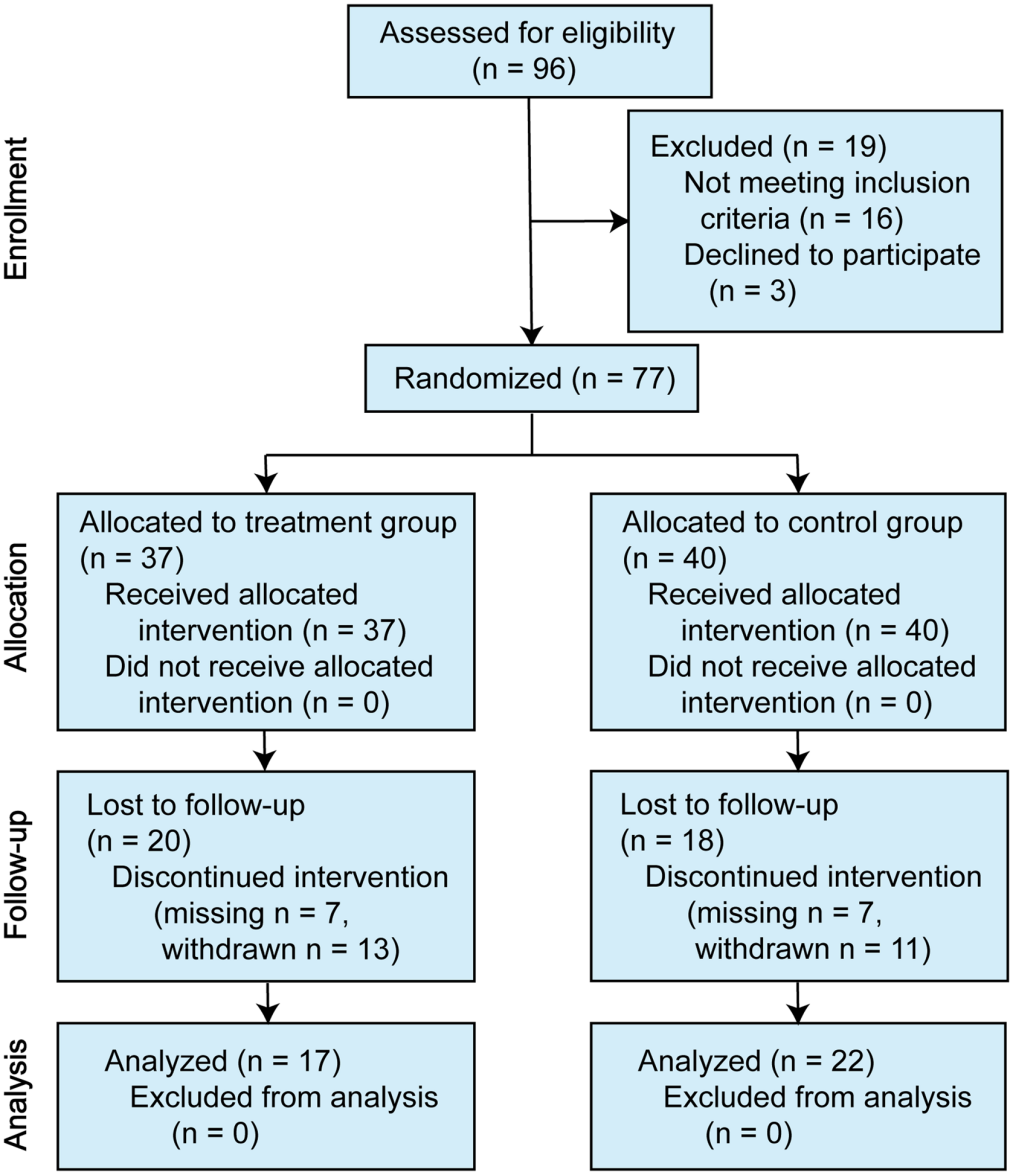
CONSORT (Consolidated Standards of Reporting Trials) diagram.

A total of 80 symptomatic individuals participated in the study. Of the three offered no reason to complete the entire assessments at baseline, leaving 77 individuals who began the drug interventions. For the SZAS-HFG group, 37 participants finished at least one assessment during the trial, and 17 participants completed the intervention and follow-ups. For the control group, 40 participants began the drug interventions, and the 24 participants completed the intervention. Thirteen participants in the SZAS-HFG group and 15 participants in the control group did not know the correct grouping after 12-week treatment. The details were shown in Figure 1.

### 3.2. Demographic and clinical information

Demographic information was shown in Table 1. We did not find any significant difference in age, gender, baseline overall psychosis symptoms (SOPS scores), baseline cognitive assessments, and overall functioning (GAF score) between the two groups. Moreover, there was also no significant difference between dropout subjects and completion subjects. The details were shown in Table 2.

**Table 1:** Demographic characteristics of UHR patients.

Characteristic	SZAS-HFG group (*N* = 37)	Control group (*N* = 40)	*t*/χ^2^	*p*
Male	19(51.35%)	25(62.50%)	χ^2^(0.57)	0.449
Age(years)^a^	20.49(3.30)	19.10(3.30)	*t*(1.84)	0.069
Education(years)^a^	12.43(2.97)	12.03(2.43)	*t*(0.66)	0.511
Dropout	20(54.05%)	18(45%)	χ^2^(1.01)	0.314
SOPS^a^	27.05(10.99)	30.27(15.03)	*t*(−1.07)	0.29
GAF^a^	47.30(8.19)	49.43(7.80)	*t*(−1.17)	0.247
TMT-A^a^	43.59(47.82)	39.48(23.15)	*t*(0.48)	0.637
HVLT^a^	26.86(5.41)	26.73(6.23)	*t*(0.11)	0.917
BVMT^a^	26.24(7.54)	26.25(6.45)	*t*(−0.004)	0.997
CPT^a^	2.81(0.71)	2.76(0.58)	*t*(0.31)	0.755

^a^Reported as mean (SD). BVMT, brief visuospatial memory test; CPT, continuous performance test; GAF, global assessment of functioning; HVLT, Hopkins verbal learning test; SOPS, scale of prodromal syndromes; TMT-A, trail making test.

**Table 2:** Demographic characteristics of UHR patients between dropout subjects and completion subjects.

Characteristic	Dropout subjects (*N* = 38)	Completion subjects (*N* = 39)	*t*/χ^2^	*p*
Age(years)^a^	21.72(5.89)	20.22(6.33)	*t*(1.06)	0.292
Education(years)^a^	12.66(2.53)	11.69(2.81)	*t*(1.62)	0.110
SOPS^a^	27.92(10.35)	30.17(15.10)	*t*(−0.74)	0.461
GAF^a^	48.56(6.22)	48.49(9.61)	*t*(0.04)	0.968
TMT-A^a^	38.29(16.86)	44.94(49.08)	*t*(−0.82)	0.416
HVLT^a^	27.34(5.33)	26.54(6.38)	*t*(0.61)	0.542
BVMT^a^	26.39(5.83)	26.38(7.89)	*t*(0.004)	0.997
CPT^a^	2.70(0.64)	2.84(0.65)	*t*(−0.95)	0.343

^a^Reported as mean (SD). BVMT, brief visuospatial memory test; CPT, continuous performance test; GAF, global assessment of functioning; HVLT, Hopkins verbal learning test; SOPS, scale of prodromal syndromes; TMT-A, trail making test-A.

### 3.3. Between group comparisons of changes in primary outcomes

**Figure 2:**
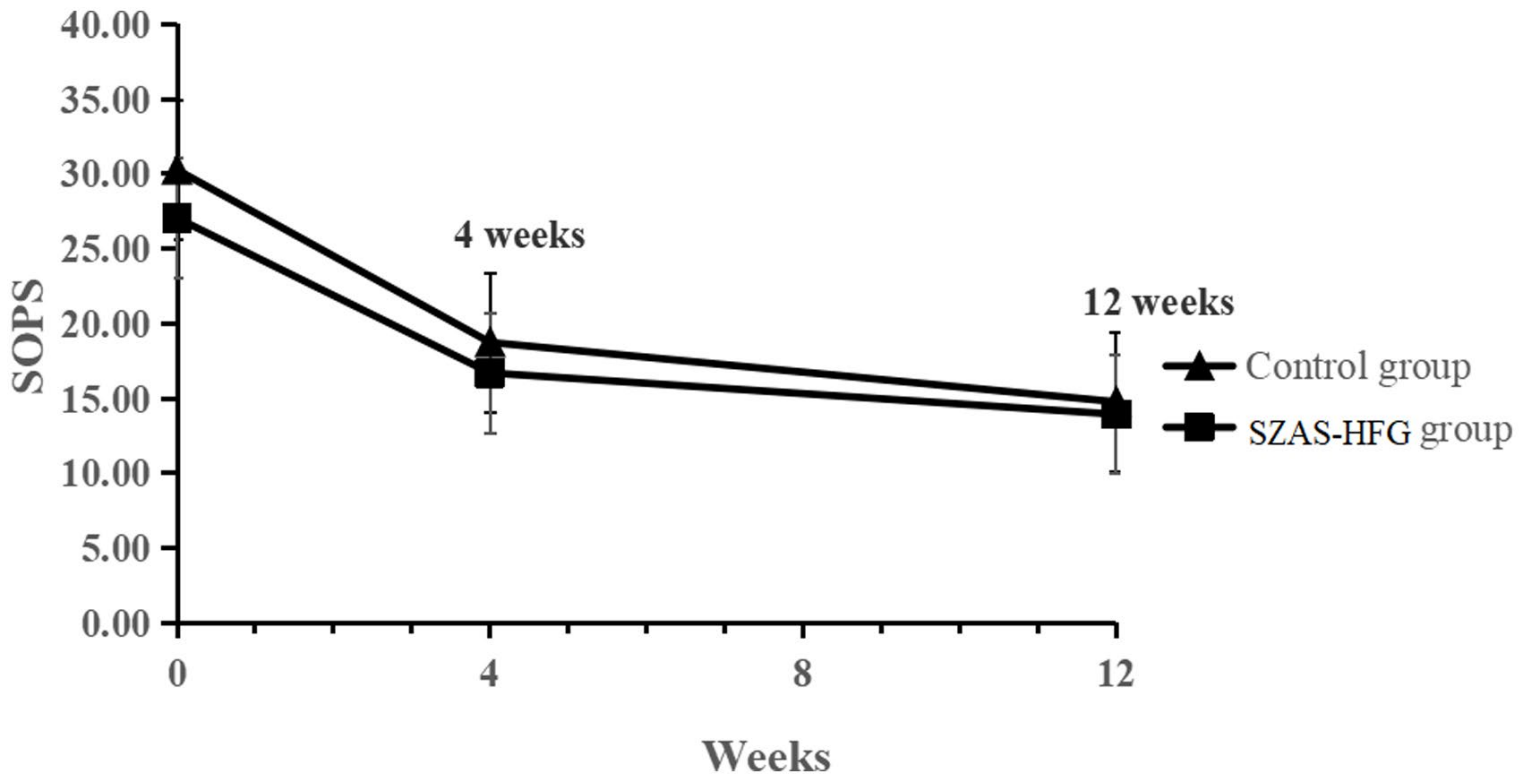
Mean SOPS total scores from baseline to 12 weeks for the two groups.

The linear mixed-effects model analysis of the SOPS score showed the significant effect of time (*F* = 116.7, *p* < 0.001), and no significant effect of group (*F* = 2.31, *p* = 0.14) and time × group interaction (*F* = 0.48, *p* = 0.062). The result indicated that both groups had a significantly decreased SOPS score and no difference between both groups (Week 4: within-group Hedges’ *g* = 0.81 versus 0.83; Week 12: within-group Hedges’ *g* = 1.16 versus 1.11). The detail was shown in Table 3 and Figure 2.

**Table 3:** Assessment of the two groups during the intervention periods on psychosis symptoms, cognition and social functioning.

		Baseline (*N* = 77)	Week 4 (*N* = 62)	Week 12 (*N* = 39)	Statistics, *p*-values		
Outcomes	Group	*M*(SD)	*M*(SD)	*M*(SD)	Time	Group	Time × Group
SOPS	SZAS-HFG	27.05(10.99)	16.70(14.61)	13.94(11.50)	<0.0001	0.27	0.91
	Control	30.28(15.04)	18.74(12.33)	14.77(11.43)			
TMT-A	SZAS-HFG	43.59(47.82)	32.42(11.17)	31.63(12.43)	0.0049	0.68	0.47
	Control	39.48(23.15)	31.66(11.39)	25.67(9.90)			
HVLT	SZAS-HFG	26.86(5.41)	29.04(5.34)	31.24(4.59)	<0.0001	0.72	0.71
	Control	26.73(6.23)	29.00(4.70)	30.05(4.85)			
BVMT	SZAS-HFG	26.24(7.54)	29.59(4.26)	28.82(5.96)	0.0002	0.84	0.99
	Control	26.25(6.45)	30.06(4.70)	29.59(6.17)			
CPT	SZAS-HFG	2.82(0.71)	3.07(0.53)	3.20(0.47)	0.24	0.02	0.20
	Control	2.79(0.55)	2.78(0.64)	2.77(0.70)			
GAF	SZAS-HFG	47.30(8.19)	59.44(14.60)	63.94(16.49)	<0.0001	0.21	0.03
	Control	49.43(7.81)	56.09(11.43)	60.86(11.05)			

BVMT, brief visuospatial memory test; CPT, continuous performance test; GAF, global assessment of functioning; HVLT, Hopkins verbal learning test; SOPS, scale of prodromal syndromes; TMT-A, trail making test.

**Figure 3:**
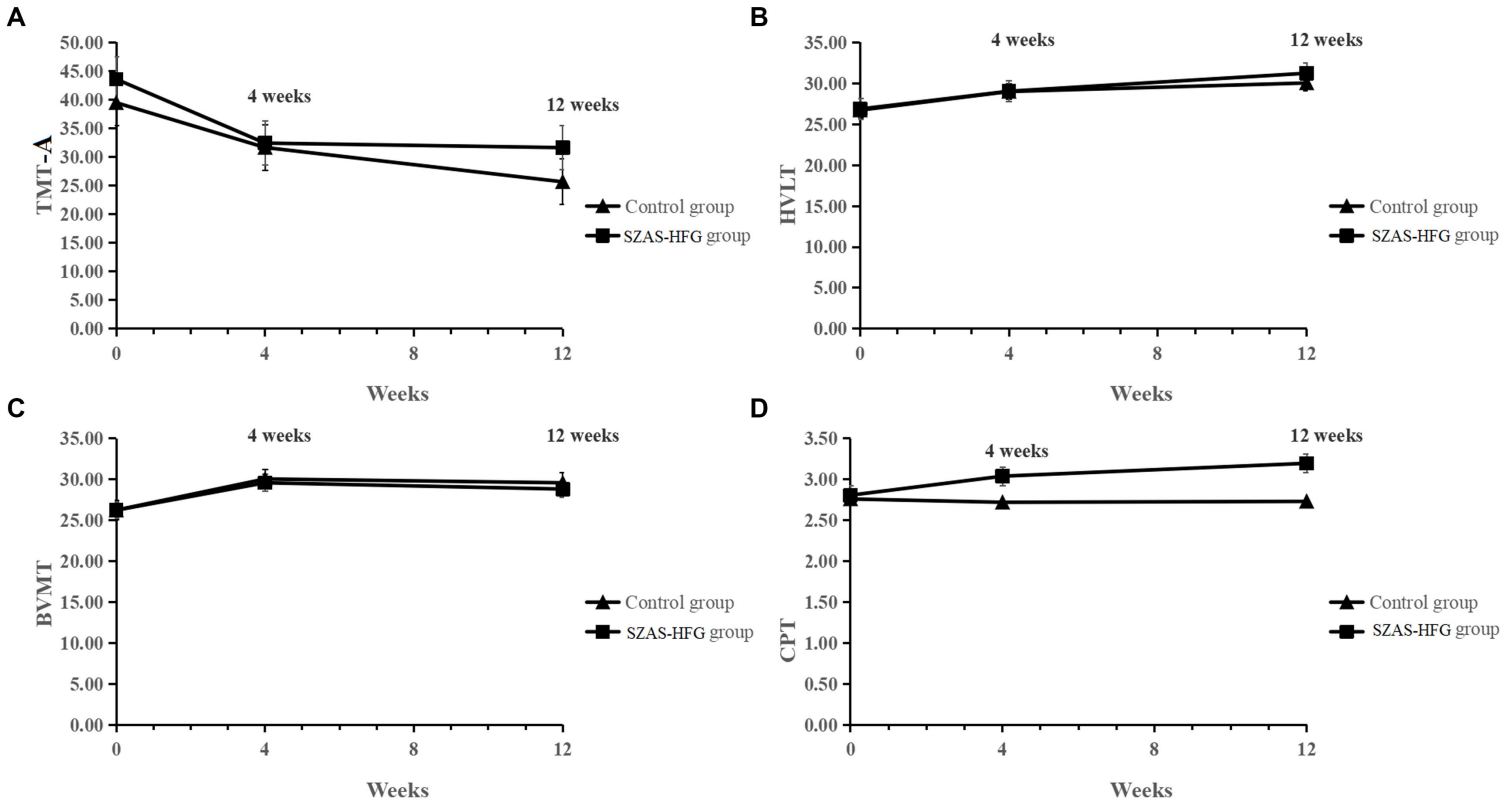
**(A)** Mean TMT-A score from baseline to 12 weeks for the two groups. **(B)** Mean HVLT score from baseline to 12 weeks for the two groups. **(C)** Mean BVMT score from baseline to 12 weeks for the two groups. **(D)** Mean CPT score from baseline to 12 weeks for the two groups.

Then, we found significant main effects of time on TMT-A (*F* = 5.76, *p* = 0.0049), HVLT (*F* = 11.42, *p* < 0.0001), and BVMT (*F* = 11.42, *p* < 0.0001). Meanwhile, there were no significant effects of interaction and group on TMT-A, HVLT, and BVMT, which showed significant increases in most domains of cognitive functions for both groups. Furthermore, there were no significant effects of time and time × group interaction on CPT, and a significant effect of group (*F* = 6.16, *p* = 0.02) on CPT between both groups (Week 4: within-group Hedges’ *g* = 0.48 versus 0.01; Week 12: within-group Hedges’ *g* = 0.82 versus 0.03). The result was shown in Table 3 and Figure 3.

### 3.4. Between group comparisons of changes in secondary outcomes

**Figure 4:**
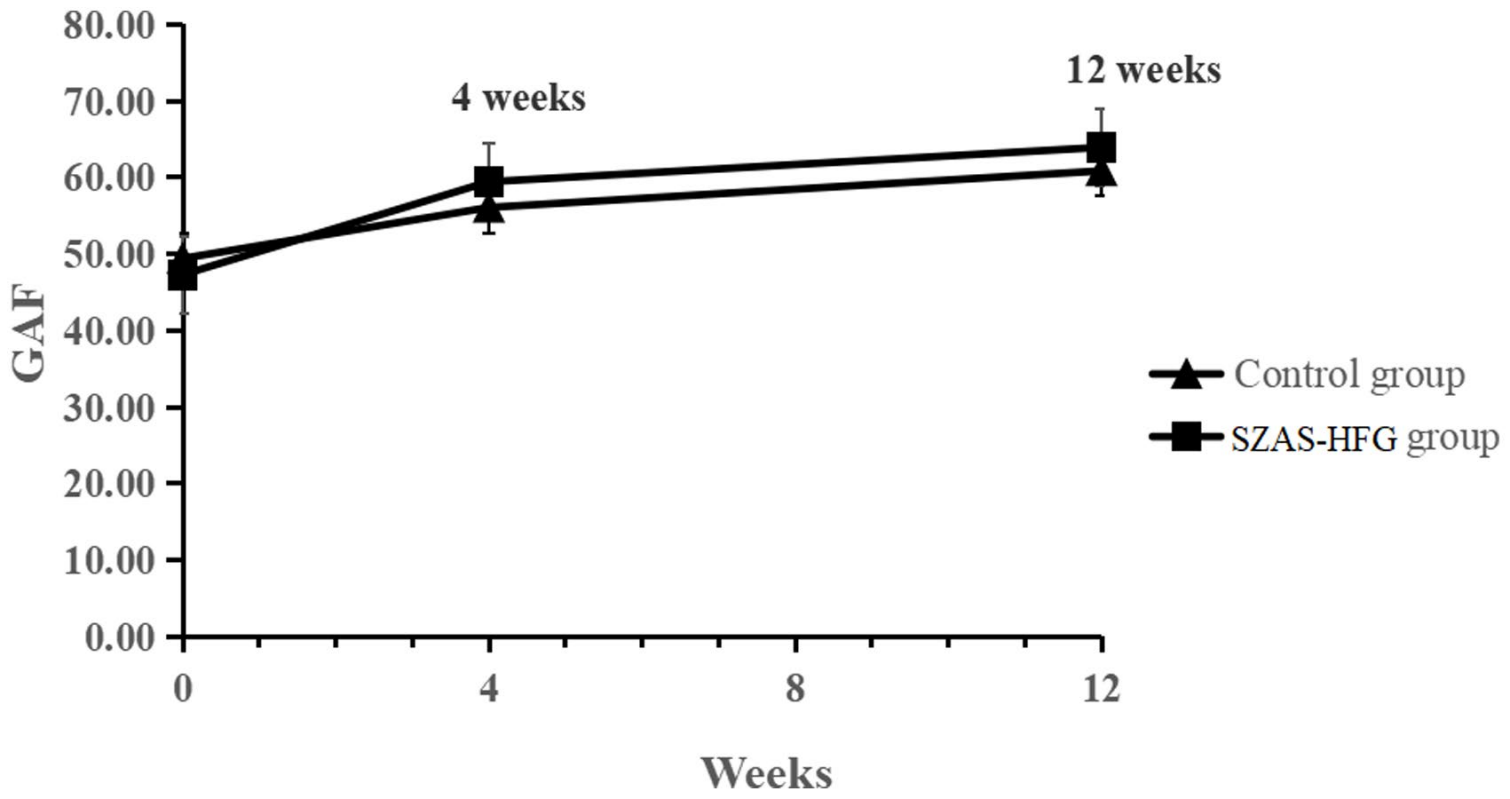
Mean GAF scores from baseline to 12 weeks for the two groups.

There was a significant effect of time on GAF, suggesting significant improvements in social functioning for both groups. The interaction effect of time × group (*F* = 3.98, *p* = 0.027) showed a significant improvement in social and occupational functioning in the SZAS-HFG group during the whole study period. GAF did not differ between both groups. The result was shown in Table 3 and Figure 4.

We also displayed the effect sizes (Hedges’ *g*) of changes within two groups at three time points Table 4. The SZAS-HFG group showed higher values of effect sizes than the control group in the HVLT, CPT, and GAF.

**Table 4:** Effect sizes of the two groups in SOPS, GAF and cognitive assessments from baseline to 12 weeks assessment.

Items	Group	Baseline vs. Week 4	Baseline vs. Week 12	Week 4 vs. Week 12
SOPS	SZAS-HFG	0.81	1.16	0.22
	Control	0.83	1.11	0.33
GAF	SZAS-HFG	1.07	1.46	0.29
	Control	0.68	1.24	0.11
TMT-A	SZAS-HFG	0.29	0.29	0.07
	Control	0.42	0.7	0.55
HVLT	SZAS-HFG	0.4	0.83	0.43
	Control	0.4	0.57	0.22
BVMT	SZAS-HFG	0.44	0.52	0.15
	Control	0.66	0.52	0.09
CPT	SZAS-HFG	0.48	0.82	0.27
	Control	0.01	0.03	0.02

Effect size: 0.20–0.49, small effect size; 0.50–0.79, moderate level; 0.80 and more, large effect size. BVMT, brief visuospatial memory test; CPT, continuous performance test; GAF, global assessment of functioning; HVLT, Hopkins verbal learning test; SOPS, scale of prodromal syndromes; SZAS-HFG, Shi-Zhen-An-Shen herbal formula granule; TMT-A, trail making test.

### 3.5. Adverse effects

We recorded adverse events at week 4 and week 12. The 10.81% UHR patients suffered at least one adverse event in the SZAS-HFG group, compared to 35% in the control group. The diarrhea was the most common adverse event in the SZAS-HFG group, while the most common adverse events in the control group were sleepiness, extrapyramidal symptoms, lassitude, and weight gain. No serious adverse events relative with SZAS-HFG or aripiprazole happened. The detail was shown in Table 5.

**Table 5:** Adverse events recorded in both groups.

Event	SZAS-HFG group (*N* = 37)		Control group (*N* = 40)	
	*N*	%	*N*	%
Subjects with at least one adverse event	4	10.81	14	35
Most common adverse events				
Sleepiness	0	0	12	30
Extrapyramidal symptoms	2	5.41	6	15
Lassitude	0	0	5	12.5
Diarrhea	4	10.81	0	0
Weight gain	1	2.7	4	10

SZAS-HFG, Shi-Zhen-An-Shen herbal formula granule.

## 4. Discussion

To our knowledge, this is the first study to explore the efficacy of Chinese herbal medicine on treating UHR patients for psychosis. After 12-week treatment, both groups showed significant effects of time on SIPS, TMT-A, HVLT, BVMT and GAF. There was a significant effect of group only on CPT. Moreover, we also found a significant effect of interaction on GAF.

The SOPS from the SIPS interview tool is developed to assess psychosis symptoms, including four components: positive symptoms, negative symptoms, disintegrative symptoms, and general symptoms (20). Previous studies have confirmed that the attenuated psychotic symptoms was associated with poorer social functioning and more sever anxiety and depressive symptoms (28), and persistent negative symptoms are associated with poor long-term social functioning and quality of life (29). Our findings showed that the score of SOPS reduced significantly in both groups after 12-week treatment. In line with previous clinical trials. Antipsychotic medications, as the main form of treatment on patients with psychosis, have been assumed to be also effective in reducing psychosis symptoms for UHR patients. One previous study on UHR patients showed the olanzapine group improved more than the placebo group in the means core for prodromal positive symptoms after 12-month treatment (30). Another study also showed a reduction in symptoms in patients treated by risperidone combined cognitive therapy (31). Meanwhile, the systematic review and meta-analysis showed the herb medicine combined with antipsychotics had more beneficial effects on psychosis symptoms than the antipsychotics alone (32). Our study on demyelinated mice exhibiting schizophrenia-like behaviors also showed the SZAS decoction could improve schizophrenia-like behaviors and reverse demyelination in the mice (33). In general, our results indicated that the SZAS-HFG had similar effect with the aripiprazole in reducing psychosis symptoms for UHR patients.

We also observed that both groups showed significant changes in verbal memory, visual learning, information processing speed and executive functions after 12-week treatment, except the attention. Nevertheless, there was no significant difference between the two groups. The finding of improving cognitive impairments was in line with previous studies on schizophrenia. It has been demonstrated that the aripiprazole treatment on recent onset schizophrenia can improve several cognitive parameters, such as delayed recall, attention, and executive functions (34). Meanwhile, it has also been reported that the Chinese herb could significantly improve executive functions in schizophrenia patients (21). Our previous study had also demonstrated that the SZAS decoction could improve the sensory gating function relative with information processing in demyelinated mice with schizophrenia-like behavior (33), and ameliorate cognitive declines in MK801-induced schizophrenia model by restoring the levels of synaptic proteins in the hippocampus (35). Moreover, some active ingredients in SZAS-HFG, including cornel iridoid glycoside and tetrahydroxystilbene glucoside, have divergent therapeutic effects on cognitive impairment and neurological defects (25, 26).

We also found that the SZAS-HFG group had fewer adverse events than the control group. The Meta-analysis on TCM decoction plus antipsychotic for schizophrenia patients has revealed TCM can reduce some side effects (32). The previous study had confirmed that the aripiprazole had definite adverse events such as weight gain and akathisia (36), which was in line with our result. According to TCM theory, the Tong Xia therapeutic method with cathartic effect of Chinese medicine was applied to treat UHR for psychosis. In hence, the diarrhea is the most common adverse event in the SZAS-HFG group, which met the treatment concept of TCM. Moreover, those with false-positive UHR individuals may be exposed to the adverse effects of antipsychotics. Considering the favorable side effect profile and the popularity of herbal in our culture, the SZAS-HFG can be promoted and applied for treating the UHR patients in China.

However, our study comes with some limitations. Firstly, in the current study, as a preliminary study, only 12-week follow up was conducted. The transition rate failed to obtain in our study. In hence, the longer-term follow-up would be needed in the future study. Secondary, the dropout rate was really high in our study. There were two possible reasons for this. One reason was that recruited patients failed to follow up on time affected by the outbreak of COVID-19. The second reason was that a number of UHR patients manifested with mild psychosis symptoms and lived far away from the hospital. And after one-month treatment, the mild psychosis symptoms were relieved, which may arise the potential biased attrition of mild cases. Thirdly, as a polit study, a placebo group would be needed to eliminate other effects such as practice effect, and results were corrected for multiple comparisons in the future.

## 5. Conclusion

In conclusion, the Chinese herbal medicine SZAS-HFG can be effective in alleviating psychosis symptoms, and improving cognitive impairments and overall as well as aripiprazole, which may provide a new approach to treat UHR for psychosis.

## Data availability statement

The raw data supporting the conclusions of this article will be made available by the authors, without undue reservation.

## Ethics statement

The studies involving human participants were reviewed and approved by Beijing Anding Hospital Ethics Committee. The patients/participants provided their written informed consent to participate in this study.

## Author contributions

All authors listed have made a substantial, direct, and intellectual contribution to the work and approved it for publication.

## Funding

This study was supported by Capital’s Funds for Health Improvement and Research (Grant no. 2018-1-2122 and 2020-4-2126), Beijing Hospitals Authority’s Ascent Plan (Grant no. DFL20191901), Training Plan for High Level Public Health Technical Talents Construction Project (Grant no. Academic Backbone-02-40) and Beijing Hospitals Authority Clinical Medicine Development of Special Funding (Grant no. ZYLX202129).

## Conflict of interest

The authors declare that the research was conducted in the absence of any commercial or financial relationships that could be construed as a potential conflict of interest.

## Publisher’s note

All claims expressed in this article are solely those of the authors and do not necessarily represent those of their affiliated organizations, or those of the publisher, the editors and the reviewers. Any product that may be evaluated in this article, or claim that may be made by its manufacturer, is not guaranteed or endorsed by the publisher.

## Footnotes

^1^ https://www.ncss.com/software/pass/
